# The aglycone of ginsenoside Rg3 enables glucagon-like peptide-1 secretion in enteroendocrine cells and alleviates hyperglycemia in type 2 diabetic mice

**DOI:** 10.1038/srep18325

**Published:** 2015-12-17

**Authors:** Ki-Suk Kim, Hea Jung Yang, In-Seung Lee, Kang-Hoon Kim, Jiyoung Park, Hyeon-Soo Jeong, Yoomi Kim, Kwang Seok Ahn, Yun-Cheol Na, Hyeung-Jin Jang

**Affiliations:** 1 Department of Biochemistry, College of Korean Medicine, Kyung Hee University, 1 Heogi-dong, Dongdaemun-gu, Seoul, 130-701 Republic of Korea; 2 Department of Pathology, College of Korean Medicine, Kyung Hee University, 1 Heogi-dong, Dongdaemun-gu, Seoul, 130-701 Republic of Korea; 3 Western Seoul Center, Korea Basic Science Institute, 150 Bugahyeon-ro, Seodaemun-gu, Seoul 120-140, Republic of Korea

## Abstract

Ginsenosides can be classified on the basis of the skeleton of their aglycones. Here, we hypothesized that the sugar moieties attached to the dammarane backbone enable binding of the ginsenosides to the sweet taste receptor, eliciting glucagon-like peptide-1 (GLP-1) secretion in the enteroendocrine L cells. Using the human enteroendocrine NCI-H716 cells, we demonstrated that 15 ginsenosides stimulate GLP-1 secretion according to the position of their sugar moieties. Through a pharmacological approach and RNA interference technique to inhibit the cellular signal cascade and using the Gαgust^−/−^ mice, we elucidated that GLP-1 secreting effect of Rg3 mediated by the sweet taste receptor mediated the signaling pathway. Rg3, a ginsenoside metabolite that transformed the structure through a steaming process, showed the strongest GLP-1 secreting effects in NCI-H716 cells and also showed an anti-hyperglycemic effect on a type 2 diabetic mouse model through increased plasma GLP-1 and plasma insulin levels during an oral glucose tolerance test. Our study reveals a novel mechanism where the sugar moieties of ginsenosides Rg3 stimulates GLP-1 secretion in enteroendocrine L cells through a sweet taste receptor-mediated signal transduction pathway and thus has an anti-hyperglycemic effect on the type 2 diabetic mouse model.

Ginsenosides are triterpenoid saponins found abundantly in Panax species (ginseng). The dammarane type family consists mostly of ginsenosides and is largely composed of a four-carbon ring structure backbone with various sugar moieties attached to the C-3 and C-20 positions and can be divided into three main groups: protopanaxadiols (PPDs), protopanaxatriols (PPTs), and ocotillol^1^,^2^. PPTs, such as Re, Rf, Rg1, and Rh1, are composed of sugar moieties attached to the α-OH at C-6 and/or β-OH at C-20 of the dammarane skeleton, while PPDs, such as Rb1, Rb2, Rc, Rd, Rg3, and Rh2, are composed of sugar moieties attached to the β-OH at C-3 or C-20^3^. Most ginsenosides have been isolated from roots, leaves, stems, fruits, and/or flower parts of *Panax ginseng*, but several types can be converted into other types of ginsenosides by a steaming process or intestinal microbiota metabolism^2^. For example, the ginsenoside Rg3, which is absent in raw ginseng, is a bioactive compound of red ginseng, a heat processed *Panax ginseng*, and PPDs, such as Rb1, Rb2, Rc, and Rd, can be converted into compound K (C-K) by the intestinal microbiota metabolism^4^,^5^.

The antidiabetic and antiobesity effects of several ginsenosides including Rb1, Re, Rb2, Rg3, and C-K have been investigated^5^,^6^,^7^,^8^,^9^. We focused on the structure of ginsenosides, which contain various sugar moieties attached to the dammarane skeleton, and hypothesized that the sugar moieties stimulate GLP-1 secretion via activation of a sweet taste receptor signaling pathway in enteroendocrine L cells.

GLP-1 is a potent anti-hyperglycemic agent, which induces glucose-dependent insulin secretion from pancreatic β cells, while suppresses glucagon secretion. For intact GLP-1 in plasma removed rapidly by renal clearance and enzyme dipeptidyl peptidase-4 (DPP-4), incretin-based treatment using DPP-4 inhibitors (gliptins) or GLP-1 receptor (GLP-1R) agonists is a frequently prescribed therapy for type 2 diabetes mellitus^10^,^11^.

The NCI-H716 cell line is derived from ascetic fluid of a 33 year old Caucasian male patient with a poorly differentiated cecal adenocalcinoma^12^. Culturing the cells with a specific extracellular matrix causes endocrine differentiation, leading the cells to express several neuroendocrine markers including chromogranin A, and on this basis they are a qualified enteroendocrine cellular model for studying the regulation of GLP-1 secretion^12^.

We have reported that enteroendocrine L cells express taste receptors and their downstream signal elements, including a specific G protein, Gα-gustducin (Gαgust), and G protein-coupled sweet and bitter taste receptors, similar to their expression in the tongue^13^,^14^. T1R3 taste receptor, which is expressed by about 10-20% of taste cells, consists of a heterodimer T1R2 that recognizes a broad spectrum of sweet taste stimuli, including natural and synthetic sugar^15^,^16^. Previous reports suggest that the intracellular signal transduction pathway activated by sugar binding to taste receptors is mediated by the activation of Gαgust and a consequent signaling cascade including phospholipase Cβ2 (PLCβ2) and inositol 1,4,5-triphosphate (IP_3_)^16^,^17^. This Gβγ-subunit mediating the signaling cascade elicits the release of Ca^2+^ from intracellular stores and subsequent Ca^2+^-dependent activation of a transient receptor potential channel M5 (TRPM5), leading to the membrane depolarization and further action potential generation in turn^18^,^19^.

In this study, we demonstrated the GLP-1 secreting effect of ginsenosides using the enteroendocrine NCI-H716 cell line. Rg3, a PPD group ginsenoside that is abundant in steamed ginseng, showed the strongest GLP-1 secreting effect. Using the cell line and Gαgust^−/−^ mice, we investigated the cellular mechanism underlying the GLP-1 secreting effect of Rg3, and using *db/db* mice, we evaluated the possibility of exploiting the effect of Rg3 as a therapeutic agent for type 2 diabetes mellitus.

## Results

**Figure 1:**
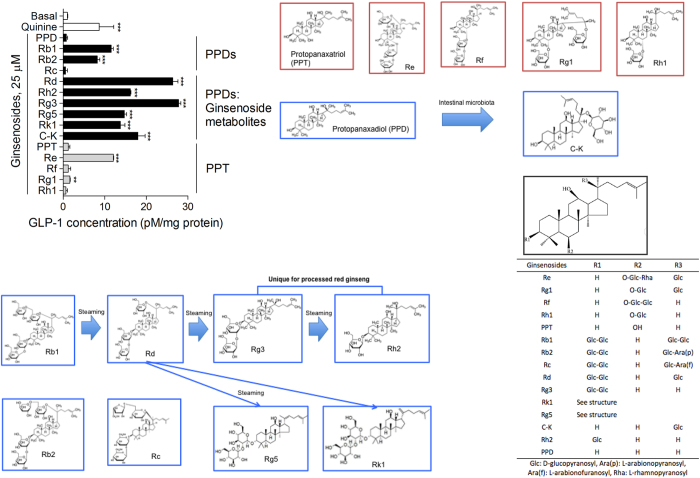
GLP-1 secreting effect of dammarane-family ginsenosides on NCI-H716 cells. The effect of each ginsenoside was compared to the GLP-1 secreting effect of the bitter tastant quinine (2 mM). Blue square, protopanaxadiol (PPD) type ginsenosides; red square, protopanaxatriol (PPT) type ginsenosides. Data are mean ± s.e.m. Statistics, Mann-Whitney U test. ^**^P < 0.01; ^***^P < 0.001 vs Basal.

To confirm our hypothesis that the ginsenosides stimulate GLP-1 secretion in enteroendocrine L cells, we treated endocrine differentiated NCI-H716 cells with 15 types of ginsenosides. We treated the PPD family, PPD, Rb1, Rb2, Rc, Rd, Rh2, Rg3, Rg5, Rk1, and C-K, and the PPT family, PPT, Re, Rf, Rg1, and Rh1^2^. We observed GLP-1 secretion in NCI-H716 cells treated with ginsenosides: Rb1, Rb2, Rd, Rg3, Rg5, Rk1, C-K, Re, and Rg1 (Fig. 1). Interestingly, Rd, Rg3, Rh2, Rg5, and Rk1, which are nearly absent in *Panax ginseng*, but abundantly found in red ginseng, showed a strong GLP-1 secreting effect in the NCI-H716 cells. The ginsenoside metabolites appear to result from deglycosylation during the steaming process^1^. The most abundantly found ginsenosides in *Panax ginseng* are Rb1, Rb2, Rc, Re, and Rg1. We observed a GLP-1 secreting effect of Rb1, Rb2, Re, and Rg1 on NCI-H716 cells, but Rc, Rf, and Rh1 had no GLP-1 secreting effect on the cells (Fig. 1). C-K, a particular ginsenoside that is only produced by intestinal microbiota metabolism, strongly stimulated GLP-1 secretion in the cells (Fig. 1). We also treated the cells with PPD and PPT but no GLP-1 secreting effect was observed (Fig. 1). The GLP-1 secreting effects of ginsenosides were compared to the effect of the bitter tastant quinine^20^.

**Figure 2:**
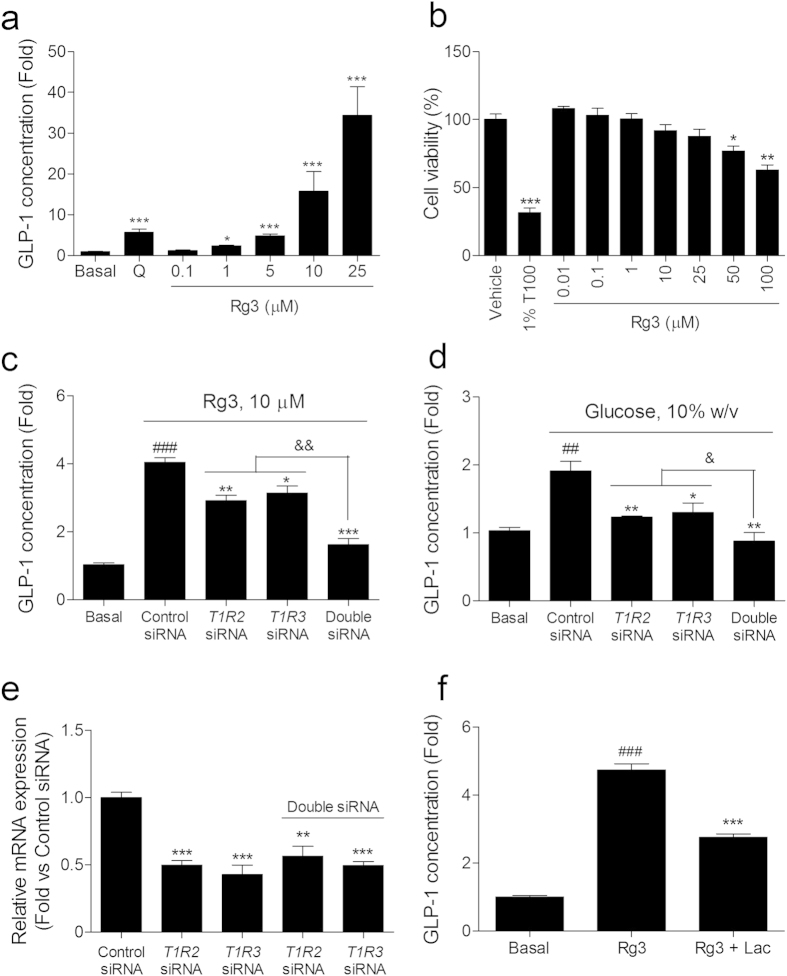
Rg3 stimulates GLP-1 secretion through the sweet taste receptor in NCI-H716 cells. (**a**) Dose-response effect of ginsenoside Rg3 on GLP-1 secretion in NCI-H716 cells. The effect was compared to the GLP-1 secreting effect of the bitter tastant quinine (Q, 2 mM). Data are mean ± s.e.m. Statistics, one-way ANOVA with Bonferroni’s post hoc test. ^*^P < 0.05; ^***^P < 0.001 vs Basal. (**b**) Effect of each dose of Rg3 on the NCI-H716 cell viability. The effect was compared to the effect of 1% triton X-100 (1%T100) on the cell viability. Data are mean ± s.e.m. Statistics, Mann-Whitney U test. ^*^P < 0.05; ^**^P < 0.01; ^***^P < 0.001 vs vehicle. (**c,d**) The GLP-1 secreting effect of ginsenoside Rg3 (**c**) and glucose (**d**) was measured in siRNA transfected NCI-H716 cells. (**e**) siRNA transfection downregulated the corresponding mRNA expression level in differentiated NCI-H716 cells. Data are mean ± s.e.m. Statistics, Mann-Whitney U test. ^##^*P* < 0.01; ^###^*P* < 0.001 vs Basal. ^*^P < 0.05; ^**^P < 0.01; ^***^P < 0.001 vs control siRNA-treated group. ^&^*P* < 0.05; ^&&^*P* < 0.01 vs *T1R2* and *T1R3* double knock-down group. (**f**) T1R3 inhibitor lactisole (Lac) inhibited the GLP-1 secreting effect of Rg3. Data are mean ± s.e.m. Statistics, Mann-Whitney U test. ^###^*P* < 0.001 vs Basal. ^***^P < 0.001 vs Rg3-treated group.

Ginsenoside Rg3 showed the strongest GLP-1 secreting effect in the NCI-H716 cells (Fig. 1). In this study, we performed further *in vitro* and *in vivo* studies using Rg3. We observed a dose-dependent GLP-1 secreting effect of Rg3 treatment in NCI-H716 cells (Fig. 2a). Rg3 did not affect the cell’s viability below a concentration of 25 μM (Fig. 2b).

To examine whether the GLP-1 secreting effect of Rg3 is mediated by sweet taste receptor activation, we transfected siRNAs targeting the *T1R2* and/or *T1R3* to the NCI-H716 cells and measured the GLP-1 levels stimulated by Rg3 treatment (Fig. 2c). We also measured glucose stimulated GLP-1 secretion to confirm our RNA interference sets (Fig. 2d). Rg3 (10 μM) showed 2-fold GLP-1 secreting effect compare to the glucose (10%, w/v). One of the *T1R2* or *T1R3* siRNA transfection partly decreased the GLP-1 secreting effects of Rg3 and glucose, respectively (Fig. 2c,d). Glucose-stimulated GLP-1 secretion was completely blocked by the *T1R2* and *T1R3* double siRNA transfection while Rg3-stimulated GLP-1 secretion slightly remained. The siRNAs accurately knocked-down their targeting mRNA expressions (Fig. 2e). A human sweet taste receptor antagonist lactisole, which have reported to block T1R3, was pre-treated NCI-H716 cells and significantly inhibited Rg3 stimulated GLP-1 secretion (Fig. 2f)^21^.

**Figure 3:**
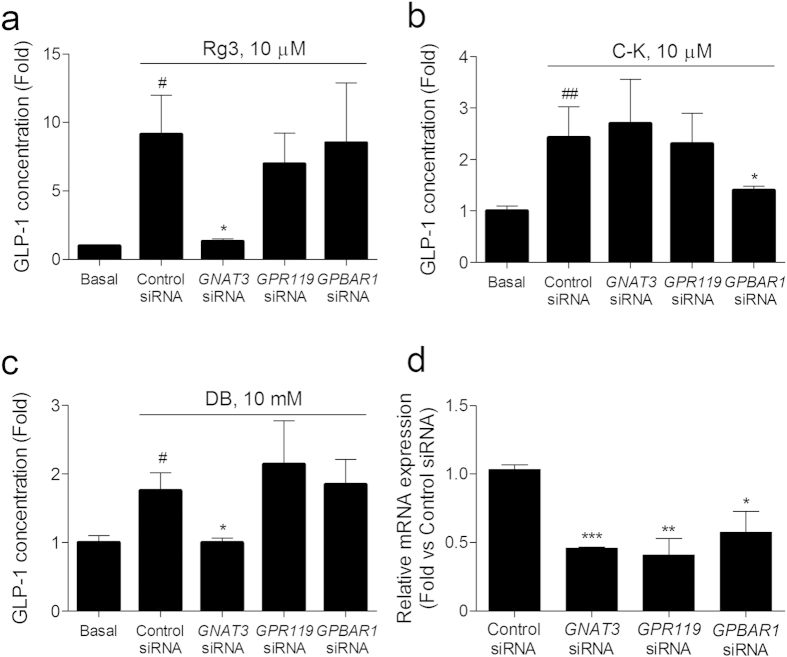
Rg3 stimulates GLP-1 secretion through the sweet taste-specific G protein in NCI-H716 cells. (**a–c**) The GLP-1 secreting effect of ginsenoside Rg3 (**a**), ginsenoside compound K (C-K; (**b**)), and bitter tastant denatonium benzoate (DB; (**c**)) was measured in siRNA transfected NCI-H716 cells. Data are mean ± s.e.m. Statistics, Mann-Whitney U test. ^#^*P* < 0.05; ^##^*P* < 0.01; ^###^*P* < 0.001 vs Basal. ^*^P < 0.05; ^***^P < 0.001 vs control siRNA-treated group. (**d**) siRNA transfection downregulated the corresponding mRNA expression level in differentiated NCI-H716 cells. Data are mean ± s.e.m. Statistics, Mann-Whitney U test. ^*^P < 0.05; ^***^P < 0.001 vs control siRNA-treated group.

We tried to identify the cellular downstream pathway of Rg3 stimulated GLP-1 secretion in the NCI-H716 cells. We blocked several GPCRs and Gαgust by transfecting siRNAs targeting the signaling molecules. Using siRNAs targeting the Gαgust gene, *GNAT3*, a cannabinoid receptor gene, *GPR119*, and a bile acid receptor 1 gene, *GPBAR1* (aliases *TGR5*), we found the GLP-1 secreting effect of Rg3 is mediated by Gαgust (Fig. 3a). We confirmed our RNA interference sets with C-K (Fig. 3b), which was reported to stimulate bile acid receptor-mediated GLP-1 secretion^5^, and also with denatonium benzoate (DB) (Fig. 3c), which was reported to stimulate Gαgust-mediated GLP-1 secretion^14^. The siRNAs accurately knocked-down their targeting mRNA expressions (Fig. 3d).

**Figure 4:**
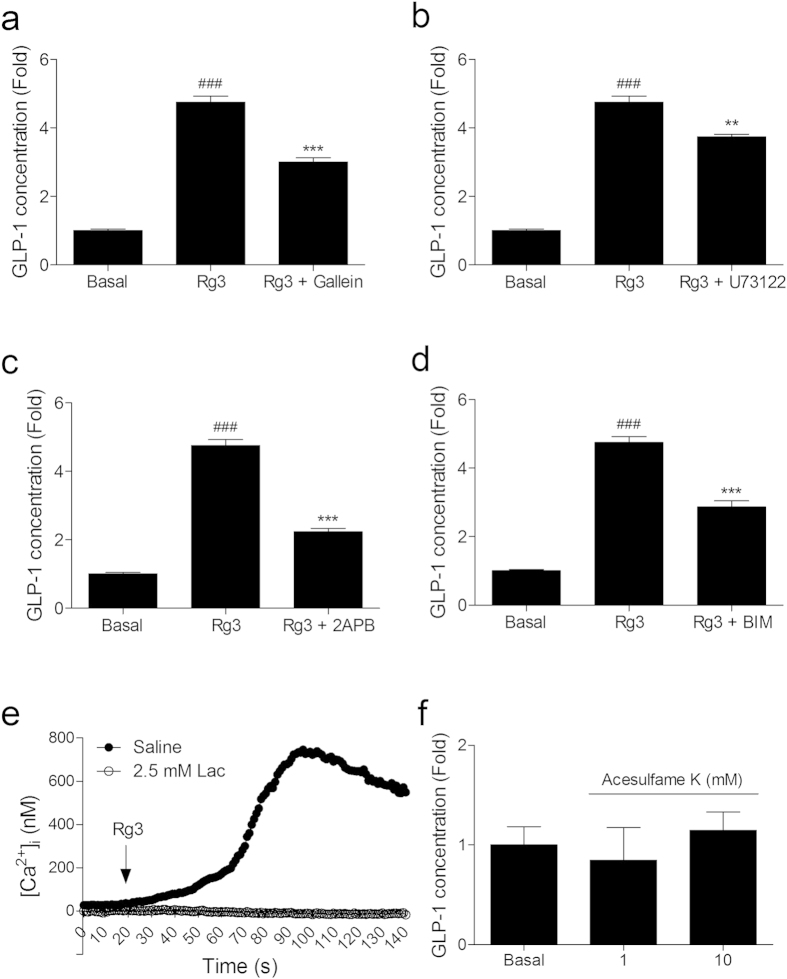
Rg3 stimulates GLP-1 secretion through the G protein βγ downstream signaling pathway. (**a–d**) The GLP-1 secreting effect of Rg3 (10 μM) on NCI-H716 cells was inhibited by blocking each downstream signal: Gβγ (gallein, 10 μM), phospholipase C (U73122, 10 μM), IP_3_ receptor (2APB, 10 μM), and protein kinase C (BIM, 10 μM). Data are mean ± s.e.m. Statistics, Mann-Whitney U test. ^###^*P* < 0.001 vs Basal. ^**^P < 0.01; ^***^P < 0.001 vs Rg3-treated group. (**e**) [Ca^2+^]_i_ increase in response to Rg3 (10 μM) treatment was blocked by the T1R3 inhibitor lactisole (Lac) in NCI-H716 cells. (**f**) An artificial sugar acesulfame K did not show GLP-1 secreting effect on NCI-H716 cells.

We then, traced the intracellular signaling pathway of Rg3 stimulated GLP-1 secretion in NCI-H716 cells. Using pathway inhibitors, Gβγ inhibitor gallein (Fig. 4a), PLC inhibitor U73122 (Fig. 4b), IP_3_ receptor antagonist 2-aminoethoxydiphenyl borate (2APB) (Fig. 4c), and PKC inhibitor bisindolylmaleimide I (BIM) (Fig. 4d), we demonstrated that the GLP-1 secreting effect of Rg3 is mediated by Gβγ-PLC-IP_3_ signaling elements, similar to the sweet taste receptor in the tongue, in the enteroendocrine NCI-H716 cells.

We also measured [Ca^2+^]_i_ levels in the NCI-H716 cells after Rg3 treatment. Enteroendocrine NCI-H716 cells were treated with Rg3 without extracellular calcium. Rg3 treatment elicited [Ca^2+^]_i_ release from the intracellular calcium store, the endoplasmic reticulum (ER), and the effect was absent in the cells that were pre-treated with the sweet taste inhibitor lactisole (Fig. 4e).

We have measured GLP-1 secreting effect of acesulfame K, a well-known artificial sweetener, on the NCI-H716 cells. However, Acesulfame K treatment did not affect the GLP-1 secreting effect of the NCI-H716 cells (Fig. 4f).

We also traced the physiological role of the Gα subunit, which has not been extensively considered in sweet taste receptor research, during Rg3 treatment. A common Gαgust-mediated signaling pathway response to the activation of G protein-coupled taste receptors, such as T1Rs or T2Rs, involves activation of phosphodiesterase (PDE) and continuous intracellular cAMP reduction^14^,^16^,^22^.

**Figure 5:**
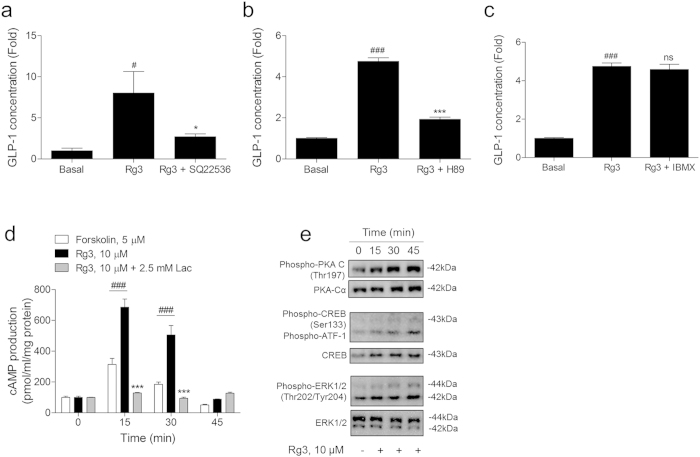
Rg3 stimulates GLP-1 secretion through the activation of adenylyl cyclase. (**a,b**) The GLP-1 secreting effect of Rg3 (10 μM) on NCI-H716 cells was inhibited by inactivation of adenylyl cyclase (SQ22536, 50 μM) and cAMP dependent protein kinase (H-89, 10 μM). (**c**) Inactivation of phosphodiesterase (IBMX, 10 μM) did not affect the GLP-1 secreting effect of Rg3 treatment. Data are mean ± s.e.m. Statistics, Mann-Whitney U test. ^#^*P* < 0.05; ^###^*P* < 0.001 vs basal. ^*^P < 0.05; ^***^P < 0.001 vs Rg3-treated group. ns, non-significant. (**d**) cAMP increasing effect of Rg3 treatment was abolished in the T1R3 inhibitor-treated cells. The cAMP increasing effect of Rg3 was measured at 15 min intervals and compared to the effect of AC activator forskolin. Data are mean ± s.e.m. Statistics, Mann-Whitney U test. ^###^*P* < 0.001 vs non-treated cell. ^***^P < 0.001 vs Rg3-treated group. (**e**) Rg3 stimulates phosphorylation of cAMP dependent protein kinase A (PKA), cAMP response element-binding protein (CREB), and extracellular signal regulated kinases (ERK) 1/2.

Surprisingly, we observed that the GLP-1 secreting effect of Rg3 is mediated by AC activation rather than PDE. Enteroendocrine NCI-H716 cells that were pre-treated with the AC inhibitor SQ22536 (Fig. 5a) or protein kinase A (PKA) inhibitor H89 (Fig. 5b) inhibited the GLP-1 secreting effect of Rg3, whereas the pan-PDE inhibitor 3-isobutyl-1-methylxanthine (IBMX) (Fig. 5c) did not affect the GLP-1 secreting effect.

We measured the Rg3 stimulated intracellular cAMP production in the NCI-H716 cells by 15 min intervals. A large amount of intracellular cAMP was measured at 15 min after Rg3 treatment and was lowered to the basal level after 45 min (Fig. 5d). The stimulatory effect of Rg3 treatment on the intracellular cAMP production was abolished in the lactisole pre-treated cells (Fig. 5d).

We then examined the phosphorylation of cAMP-dependently activated signal transduction elements. PKA, a cAMP-dependent protein kinase, CREB, a cAMP-responsive transcription factor, and extracellular signal regulated kinase (ERK) 1/2, a regulator of CREB function, were phosphorylated after Rg3 treatment (Fig. 5e).

**Figure 6:**
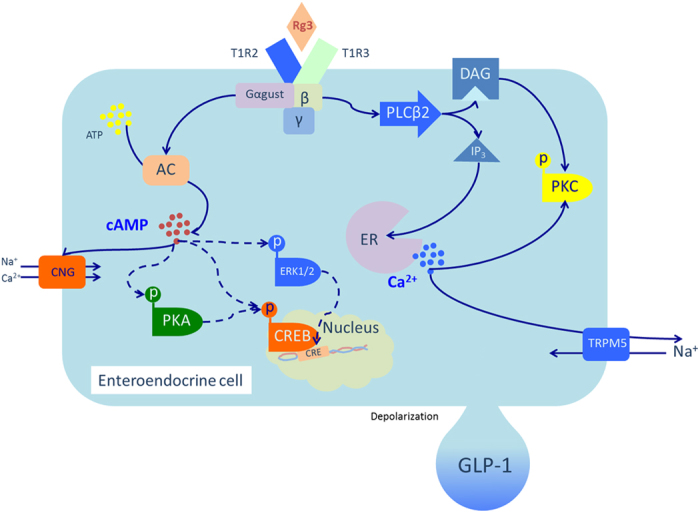
Schema of putative molecular pathways mediated by sweet taste receptor activation in NCI-H716 cell upon the Rg3 treatment.

We provide a figure that suggests the intracellular signal transduction event mediated by sweet taste receptor activation in NCI-H716 cells upon the Rg3 treatment (Fig. 6).

**Figure 7:**
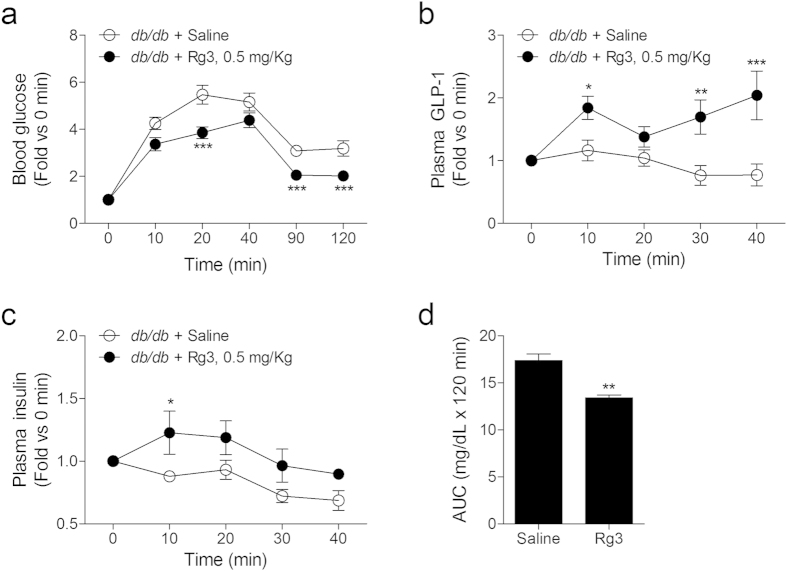
Rg3 regulates blood glucose levels by increasing plasma GLP-1 and plasma insulin secretion in *db/db* mice. (**a**) Blood glucose lowering effect of Rg3 treatment in *db/db* mice during the OGTT. Data are mean ± s.e.m.; *n* = 8. Statistics, Mann-Whitney U test. ^***^P < 0.001 vs saline-treated group. (**b,c**) Plasma GLP-1 and plasma insulin increased by Rg3 treatment in the *db/db* mice after glucose gavage. Data are mean ± s.e.m.; *n* = 5–6. Statistics, Mann-Whitney U test. ^*^P < 0.05; ^***^P < 0.001 vs saline-treated group. (**d**) Area under the curve (AUC) of the blood glucose variance during the OGTT. Data are mean ± s.e.m.; *n* = 8. Statistics, Mann-Whitney U test. ^**^P < 0.01 vs saline-treated group.

Subsequently, we performed oral glucose tolerance test (OGTT) using *db/db* mice and observed the effect of oral administration of Rg3 on hyperglycemia. The fasting glucose levels of *db/db* mice ranged from 200–300 mg/dl. Comparing to the saline-treated *db/db* mouse group, the Rg3-treated group showed a delayed increase in blood glucose level at 20 min after the treatment, and showed lowered blood glucose levels at 90 and 120 min after the treatment (Fig. 7a).

We also performed OGTT with the same *db/db* mouse groups, and collected the blood to measure the effect of Rg3 administration on the plasma GLP-1 and plasma insulin levels. Fasting plasma GLP-1 levels of *db/db* mice ranged from 70–190 pg/ml and the plasma GLP-1 level in the Rg3-treated mouse group was increased about 2-fold after 10 min during the OGTT (Fig. 7b). Fasting plasma insulin levels ranged from 4793–12982 pg/ml and the Rg3 treatment also increased the plasma insulin level about 1.3-fold after 10 min during the OGTT (Fig. 7c). The area under the curve shows the variation in the blood glucose levels of each *db/db* mouse group during OGTT (Fig. 7d).

**Figure 8:**
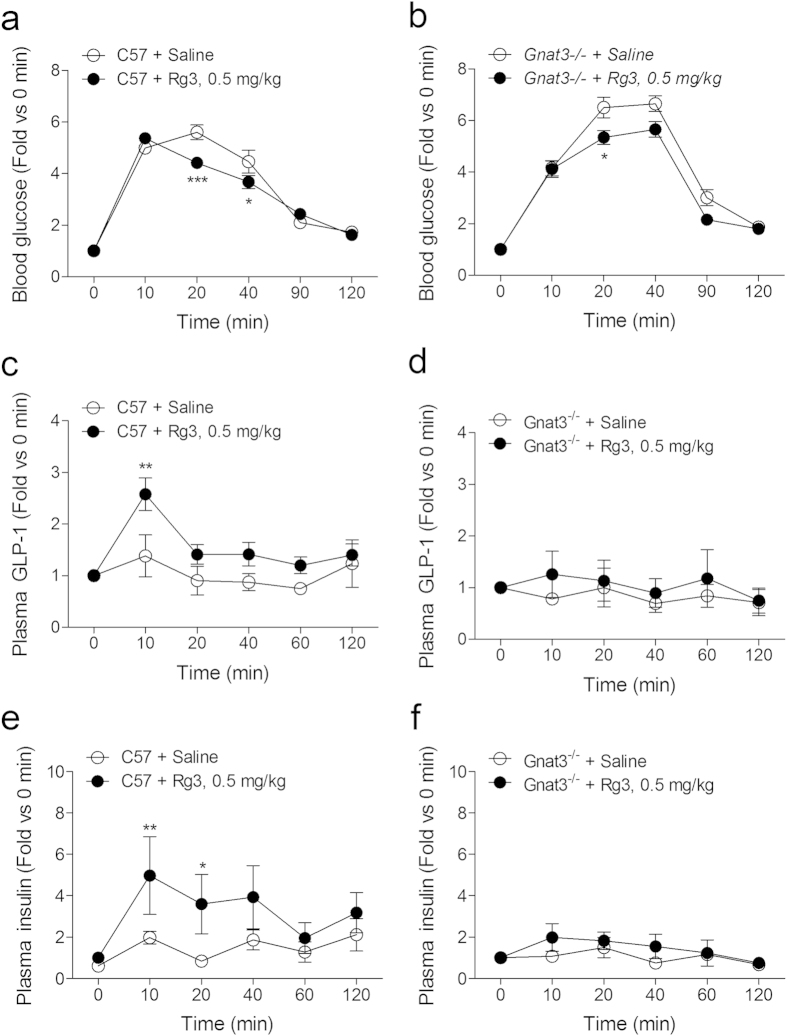
Plasma GLP-1 and plasma insulin increasing effect of Rg3 was abolished in Gαgust^−/−^ mice. (**a,b**) Blood glucose lowering effect of Rg3 treatment in C57BL/6 (C57) mice (**a**) and Gαgust^−/−^ mice (**b**). Data are mean ± s.e.m.; *n* = 6. Statistics, Mann-Whitney U test. ^*^P < 0.05; ^***^P < 0.001 vs saline-treated group. (**c–f**) The effect of Rg3 treatment on the plasma GLP-1 and plasma insulin levels in the C57 mice (**c,e**) after glucose gavage was abolished in the Gαgust^−/−^ mice (**d,f**). Data are mean ± s.e.m.; *n* = 6. Statistics, Mann-Whitney U test. ^**^P < 0.01; ^***^P < 0.001 vs saline-treated group.

We performed the same experiments using Gαgust^−/−^ mice to elucidate the involvement of Gαgust in the anti-hyperglycemic effect of Rg3 administration. We observed lowered blood glucose levels in both C57- and Gαgust^−/−^ mouse groups after 20 min of Rg3 administration during OGTT (Fig. 8a,b). However, the increased plasma GLP-1 level observed in the Rg3-treated C57 mouse group (Fig. 8c) was abolished in the Gαgust^−/−^ mouse group during the OGTT (Fig. 8d). Moreover, the stimulatory effect of Rg3 administration observed in the Rg3-treated C57 mouse group (Fig. 8e) was also abolished in the Gαgust^−/−^ mouse group at the same time (Fig. 8f).

## Discussion

*Panax ginseng* is considered one of the most valuable medicinal plants in Asia and is largely consumed throughout the world. Ginsenosides, the saponins found in all parts of the ginseng plant, have been investigated for their various pharmacological effects on hyperglycemia, weight gain, neuroprotection, tumor cell growth, and hypertension. Regarding the diverse pharmacological aspects of ginsenosides, Attle *et al.* explained that the ginsenosides share structural similarities with steroid hormones, especially progesterone and pregnanolone, and thereby have numerous physiological activities^23^. Structural diversity including type-, number-, and site of attachment of sugar moieties also contributes to the diverse pharmacological effects of ginsenosides^23^. This structural diversity can be amplified through a steaming process and intestinal microbiota metabolism.

We focused on the chemical structure of dammarane family ginsenosides having various sugar moieties with their carbon-ring backbone and hypothesized that these sugar moieties act like ligands for the sweet taste receptor.

We demonstrated the GLP-1 secreting effect of 15-dammarane family ginsenosides in human enteroendocrine NCI-H716 cells. PPDs that have sugar moieties attached at C-3 and C-20 showed a GLP-1 secreting effect on the NCI-H716 cells. On the other hand, PPTs that have sugar moieties attached at C-6 did not show the GLP-1 secreting effect on the NCI-H716 cells while the ginsenoside Re and Rg1, which has a glucose residue at C-20, showed a moderate GLP-1 secreting effect. From the obtained results, we assume that the sugar moieties attached at the C-3 and/or C-20 contribute to the binding affinity of the dammarane family ginsenosides to the sweet taste receptor.

Interestingly, ginsenoside metabolites, the chemical structure of which was transformed through a steaming process or intestinal microbiota metabolism, showed the strongest GLP-1 secreting effect. Ginsenoside Rg3 showed the strongest GLP-1 secreting effect and we traced the intracellular mechanism using enteroendocrine NCI-H716 cells.

Structurally, Rg3 is a PPD with two D-glycopyranosyl moieties. Thus we have assumed that the sugar moieties of ginsenoside Rg3 provide a binding motif to the sweet taste receptor expressed on the enteroendocrine L cells. GLP-1 secreting effect of Rg3 was significantly decreased in each T1R2 or T1R3 siRNA transfected NCI-H716 cells, respectively. But the effect was not removed completely even both the siRNAs transfection. A considerable GLP-1 secretion in the both siRNAs transfected cells response to Rg3 stimuli suggesting the existence of multiple receptors for Rg3.

A recent study reported that a ginsenoside metabolite C-K stimulates GLP-1 secretion in NCI-H716 cells via binding of a bile acid receptor^5^. We assumed that the dammarane backbone of Rg3 may contribute a binding motif to the other receptors, such as a bile acid receptor or a cannabinoid receptor, and transfected the corresponding siRNAs to abolish the mRNA expression. The GLP-1 secreting effect of Rg3 was not mediated by the cannabinoid- or bile acid receptors. Nevertheless, the GLP-1 secreting effect of Rg3 appears to be dependent on the Gαgust. Therefore, we assume participation of one or more bitter taste receptor activation in the GLP-1 secreting effect of Rg3 alongside sweet taste receptor.

We traced a common sweet taste modulatory cellular pathway, which is mediated by the Gβ_3_γ_13_-PLCβ2-IP3 signal cascade. Through pharmacological approaches using corresponding inhibitors or antagonist, we determined that the GLP-1 secreting effect of Rg3 is mediated by the signal cascade, but also found that a considerable GLP-1 response remained.

Gαgust is expected to cause activation of PDE and thus decrease intracellular cAMP levels^24^. Indeed, we observed that the bitter tastant DB decreased intracellular cAMP levels during its GLP-1 secreting event responding to Gαgust activation^14^.

However, similar to studies showing that sugars increase intracellular cAMP levels, we also have found that Rg3 increases intracellular cAMP levels. We further showed the involvement of enzyme AC, which produces cAMP in response to the stimuli, in the GLP-1 secreting effect of Rg3 instead of PDE. Moreover, phosphorylations of PKA and CREB, which are activated in response to the increased intracellular cAMP level, are involved in the GLP-1 secreting effect of Rg3. PKA is also involved in the GLP-1 secreting effect of Rg3.

ERK1/2, a mitogen-activated protein kinase (MAPK), is known to activate various transcription factors including CREB, in response to diverse extracellular stimuli such as forskolin^25^.

One of the interesting results in our study is that acesulfame K, an artificial sugar, did not affect the GLP-1 secreting effect of NCI-H716 cells. Since the expression of sweet taste receptors have been found in the enteroendocrine L cells along with its signal transduction elements artificial sugars had been convinced that they are able to activate the sweet taste receptors in the L cells as they do in the lingual tissues^13^. In contrast to the *in vitro* studies that show GLP-1 secreting effect of artificial sugars in the human and mouse enteroendocrine cells, *in vivo* studies using healthy human subjects failed to show the effects of artificial sugars on the GLP-1 secretion^26^,^27^,^28^.

However, a human study demonstrated that the sweet taste receptor inhibitor drastically blocked the GLP-1 and PYY secreting effect of glucose^29^. Perhaps, the GLP-1 and PYY secreting effect via activation of sweet taste receptor in the enteroendocrine L cells depends on the structural analogy to glucose than the sweetness itself.

Our *in vivo* study elucidated the effects of ginsenoside Rg3 administration on hyperglycemia in type 2 diabetic mice. The therapeutic effects of Rg3 against metabolic disorder, such as obesity and hyperglycemia have been demonstrated. Park *et al.* reported enhanced glucose-stimulated insulin secretion via AMP-activated protein kinase (AMPK) activation upon Rg3 treatment in a hamster pancreatic β cell line^7^. The effects of Rg3 on AMPK activation suppressed adipocyte differentiation in mouse 3T3-L1 adipocyte and improved glucose uptake in the rat L6 myocyte^30^,^31^. Therefore, Rg3 has direct glucose lowering effect on hyperglycemia through enhanced glucose uptake activity in the myoblast and also has an indirect effect through stimulation of insulin secretion in the pancreatic β cell.

In this study, Rg3 stimulated GLP-1 secretion by activating sweet taste receptor-and Gαgust-mediated signal transduction cascade in enteroendocrine cells and increased plasma GLP-1 and plasma insulin levels in *db/db* mice after glucose gavage. Using Gαgust^−/−^ mice, we showed the stimulatory effect of Rg3 on plasma GLP-1 and that plasma insulin is Gαgust-dependent. The slightly lowered blood glucose level after Rg3 administration observed in the Gαgust^−/−^ mice appears to demonstrate a direct effect of Rg3 on the blood glucose regulation^7^.

Our study elucidates a novel mechanism underlying the anti-diabetic effects of ginsenoside Rg3. The sugar moieties of Rg3 and other dammarane family ginsenosides enables their binding to the sweet taste receptor to stimulate GLP-1 secretion in intestinal L cells. The GLP-1 secreting effect lowers the blood glucose levels through insulinotropic action. Considering the diverse benefits of GLP-1 on hyperglycemia, food intake, and even β cell function, Rg3 has possibility to be developed as a novel therapeutic agent for type 2 diabetes and obesity.

## Methods

### Chemicals

All ginsenosides were kindly provided by Dr. Kwang-Seok Ahn (Department of Pathology, College of Korean Medicine, Kyung Hee University, Seoul, South Korea). Rg3, DB, metformin hydrochloride, acesulfame K, D-glucose, forskolin, and the GPCR pathway inhibitors; U73122, BIM, H-89, IBMX were purchased from Sigma-Aldrich (St. Louis, MO, USA). Gallein, 2APB, and SQ22536 were purchased from Santa Cruz Biotechnology (Santa Cruz, CA, USA). Lactisole was purchased from Endeavour Speciality Chemicals (Daventry, UK).

### Cell culture

Human NCI-H716 cells were obtained from the Korean Cell Line Bank (KCLB^®^, Seoul, South Korea). Cells were maintained in RPMI 1640 (Lonza, Walkersville, MD, USA) supplemented with 10% fetal bovine serum (FBS; Lonza). For endocrine differentiation, the cells were plated in matrigel (BD Bioscience, Bedford, MA, USA)-precoated 24-well plates at 5 × 10^5^ cells per well. The cells were incubated for 48 h in a humidified CO_2_ incubator as described previously^13^,^14^.

### GLP-1 ELISA

The endocrine differentiated cell media was replaced with PBS containing 1 mM calcium chloride and different drug concentrations. The GPCR pathway inhibitors were pre-incubated for 30 min before the drug treatment. After incubation for 1 h in a CO_2_ incubator, GLP-1 concentration was measured as previously described^14^. An active GLP-1 ELISA (EMD Millipore, Billerica, MA, USA) was performed as described in the manufacturer’s instructions. The active GLP-1 concentrations in each sample were measured using a Fluoroskan Ascent FL machine (Thermo Fishwer Scientific, Vantaa, Finland). The lowest level of active GLP-1 that can be detected by the GLP-1 assay is 2 pM.

### Cell viability assay

A cell viability assay was performed using 3-(4,5-dimethylthiazol-2-yl)-2,5-diphenyltetrazolium bromide (MTT; Invitrogen, Carlsbad, CA, USA) according to the manufacturer’s instructions.

### siRNA transfection

siRNA duplexes for *T1R2*, *T1R3*, *GNAT3* (Gαgust gene), *GPR119* and *GPBAR1* were synthesized by Bioneer (Bioneer Co., Daejeon, South Korea). The information for each siRNA was provided online (Supplementary Table S1). A scrambled negative control siRNA was purchased from Bioneer. Endocrine differentiated NCI-H716 cells were transfected with the siRNA duplexes using Lipofectamine RNAiMAX reagent (Invitrogen).

### Real-time quantitative PCR

The expression of *T1R2*, *T1R3*, *GNAT3*, *GPR119*, and *GPBAR1* after siRNA transfection was determined using a StepOne real-time PCR instrument (Applied Biosystems, Foster City, CA, USA). Total RNA isolation and subsequent cDNA hybridization were performed as previously described^32^. The expression levels of *T1R2*, *T1R3*, *GNAT3*, *GPR119*, and *GPBAR1* in each type of siRNA-transfected cell were compared with the corresponding levels in the negative control siRNA-transfected cells, and the the 2^−ΔΔCt^ values were determined^33^. *GAPDH* was used as an endogenous control. The sense and antisense sequence of each primer were provided online (Supplementary 02).

### Calcium imaging

NCI-H716 cells were seeded on a clear-bottom 96-well black plate (Corning, Tewksbury, MA, USA). After differentiation, the medium was replaced with PBS and the mixture was incubated for 30 min with fura-2 AM dye as described previously^14^,^34^. After 30 min, the medium was replaced with saline with or without 2.5 mM lactisole and incubated for a further 30 min. [Ca^2+^]_i_ were observed with a Nikon Eclipse TS 100 fluorescence imaging system (Nikon, Melville, NY, USA), and quantified and visualized with InCyt Im2 software (University of Cincinnati, Cincinnati, OH, USA). The number of cells observed was 10–20 per well.

### cAMP ELISA

Endocrine differentiated NCI-H716 cells were incubated with Rg3 or forskolin. The drug-treated cells were collected at 15 min intervals. Lactisole was pre-treated prior to Rg3 treatment. The collected cells were lysed using 0.1 M HCl, and the intracellular cAMP was assayed using ELISA (Enzo Life Sciences, Farmingdale, NY, USA) according to the manufacturer’s instructions. The results were normalized to the protein concentration.

### Immunobloting

Cells were lysed with cell lysis buffer (1% NP-40, 150 mM NaCl, 20 mM Tris-HCl, 1 mM EDTA, 1 mM EGTA, and a proteinase inhibitor cocktail). Total protein (5 μg) was loaded into each well and immunoblotting was performed as described previously^13^,^14^,^35^. Individual proteins were detected with primary antibodies against the phosphorylated form of PKA-C (1:1000 dilution), total PKA-C (1:1,000 dilutions), the phosphorylated form of cAMP-response element-binding protein (CREB) (1:3,000 dilution), total CREB (1:3,000 dilution), the phosphorylated form of ERK 1/2 (1: 3,000 dilution), and total ERK 1/2 (1:3,000 dilution). All primary antibodies were purchased from Cell Signaling (Danvers, MA, USA). After incubation with horseradish peroxidase conjugated secondary antibodies (Santa Cruz Biotechnologies), signals were visualized by enhanced chemiluminescence (Daeil Lab Service Co., Ltd. Seoul, South Korea).

### Animals

All experiments were performed in accordance with the guidelines and the regulations of the Institutional Ethical Committee of Kyung Hee University Institutional Animal Care and Use. All animal study protocols were approved by the Institutional Animal Care and Use Committee of Kyung Hee University (KHUASP(SE)-12-032). Eight-week old male *db/db* mice and C57BL/6 mice were purchased from Daehan Biolink (DBL, Eumseoung-gun, Chungcheongbuk-do, South Korea). The original mating pairs of Gαgust^−/−^ mice were kindly provided by Dr. Robert F. Margolskee (Monell Chemical Senses Center, Philadelphia, PA, USA). Gαgust^−/−^ mice, a homozygous null for the *Gnat3* allele, were created using homologous recombination in 129/Sv background embryonic stem (W9.5) cells and genotyped as previously described^36^. All animals were acclimated for one week before the experiment. The animals were housed in a room with a light-dark cycle of 12 h at a temperature ranging from 21–23 °C and moderate humidity (55–60%). Food and water were provided *ad libitum*.

### OGTT

Mice were fasted for 18 h before the OGTT. Each mouse group was orally administered saline or Rg3 (0.5 mg/Kg) just before glucose gavage (5 g/Kg). The blood glucose was measured from the tail vein using an Accu-Check Performa system (Roche Diagnostics, Mannheim, Germany) at 6 time points: 0 (before glucose gavage), 10 (after glucose gavage), 20, 40, 90, and 120 min.

### Plasma GLP-1 and plasma insulin assay

To prevent hypotensive shock, all mice were allowed to rest for one week. Mice were fasted for 18 h before the experiments. Each mouse group was orally administered saline or Rg3 (0.5 mg/Kg) just before the glucose gavage (2 g/Kg). The collected blood from the tail veins was immediately transferred into EDTA-coated microcentrifuge tubes containing a dipiptidyl peptidase IV inhibitor (EMD Millipore) and a protease inhibitor cocktail (Roche Diagnostics). The collected blood samples were centrifuged at 1,000 × *g* for 20 min at 4 °C, and the plasma was carefully separated into fresh tubes. A multiplex assay (Mouse Diabetes panel: total GLP-1 and insulin; Bio-Rad) was performed as described in the manufacturer’s instructions. The total GLP-1 and insulin concentrations in each sample were measured using a Bio-Plex MAGPIX Multiplex reader (Bio-Rad). The results were analyzed with Bio-Plex Manager software (Bio-Rad).

### Statistical analysis

GraphPad Prism 5 software (GraphPad Software, San Diego, CA, USA) was used for the statistical analysis of the experimental results. The results from the GLP-1 and cAMP ELISA represent at least three separate experiments performed in quadruplicate. The statistical significance of each ELISA and area under the curve (AUC) graph was measured using Mann-Whitney U test (one-tailed) or a one-way ANOVA with Bonferroni’s post hoc test. For OGTT and the mouse plasma hormone studies, Mann-Whitney U test was done for the inter group comparison at each time point. Five to eight mice per group were used for the *in vivo* studies. The *in vivo* studies were performed at least twice.

## Additional Information

**How to cite this article**: Kim, K.-S. *et al.* The aglycone of ginsenoside Rg3 enables glucagon-like peptide-1 secretion in enteroendocrine cells and alleviates hyperglycemia in type 2 diabetic mice. *Sci. Rep.* **5**, 18325; doi: 10.1038/srep18325 (2015).

## Supplementary Material

Supplementary Information
