# Supplementary material for: The aglycone of ginsenoside Rg3 enables glucagon-like peptide-1 secretion in enteroendocrine cells and alleviates hyperglycemia in type 2 diabetic mice
Source: Sci Rep. 2015 Dec 17;5:18325. doi: 10.1038/srep18325 (PMC4682129; doi:10.1038/srep18325)
Supplement: Supplementary Information [file srep18325-s1.pdf]

Supplementary Information

The aglycone of ginsenoside Rg3 enables glucagon-like peptide-1 secretion in enteroendocrine cells and alleviates hyperglycemia in type 2 diabetic mice.

Ki-Suk Kim[1], Hea Jung Yang[1], In-Seung Lee[1], Kang-Hoon Kim[1], Jiyoung Park[1], Hyeon-Soo Jeong[1], Yoomi Kim[1,3], Kwang Seok Ahn[2], Yun-Cheol Na[3] & Hyeung-Jin Jang[1,*]

[1]Department of Biochemistry and [2]Department of Pathology, College of Korean Medicine, Kyung Hee University, 1 Heogi-dong, Dongdaemun-gu, Seoul, 130-701 Republic of Korea

[3]Western Seoul Center, Korea Basic Science Institute, 150 Bugahyeon-ro, Seodaemun-gu, Seoul 120-140, Republic of Korea

[*]Corresponding. hjjang@khu.ac.kr

Supplementary Table S1. Sequence information for siRNA duplexes.

Gene	Forward	Reverse
T1R2	GUGAAGGUGAUAGGCUACA (dTdT)	UGUAGCCUAUCACCUUCAC (dTdT)
T1R3	CUGUCUACGCAGCUGUGUA (dTdT)	UACACAGCUGCGUAGACAG (dTdT)
GNAT3	CGAGUGAAAACGACUGGAA (dTdT)	UUCCAGUCGUUUUCACUCG (dTdT)
GPR119	CUCAUUUGGAGUGAUCCUU (dTdT)	AAGGAUCACUCCAAAUGAG (dTdT)
GPBAR1	GUCUACUUGGCUCCCAACU (dTdT)	AGUUGGGAGCCAAGUAGAC (dTdT)

The product name of each siRNAs: *T1R2* siRNA, 1148235 duplex; T1R3 siRNA, 1148240; *GNAT3* siRNA, 1175215; *GPR119*, 1064119; *GPBAR1*, 1063760.

Supplementary Table S2. Sequence information for real-time PCR primers.

Gene	Forward	Reverse
GAPDH	GCCACATCGCTCAGACACC	CCCAATACGACCAAATCCGT
T1R2	TCCCTATGTCCATGTGTTCCAA	TGCCCACAGGCTTCTTCTTT
T1R3	CGTGAGCGCAGGGCTAAA	ACGCTATACACAGCTGCGTAGACA
GNAT3	CTATGACATGGTCCTCGTGGAA	GATACTGTTGAACAGGTGAAGGCTT
GPR119	TCTCGGCCCACACAGAAGA	GCTGCGGAGGAAGTGACAA
GPBAR1	CCGGCCCCAGCATTG	TTTAGTTCAAGTCCAGGTCGACACT
